# Editorial: Monoclonal gammopathies of clinical significance: Clinical and therapeutic implications

**DOI:** 10.3389/fendo.2023.1107283

**Published:** 2023-01-17

**Authors:** Chen Wang, Chuanhui Xu, Jian Li

**Affiliations:** ^1^ Department of Internal Medicine, Morsani College of Medicine, University of South Florida, Tampa, FL, United States; ^2^ Department of Rheumatology, Allergy and Immunology, Tan Tock Seng Hospital, Singapore, Singapore; ^3^ Department of Hematology, Peking Union Medical College Hospital, Beijing, China

**Keywords:** monoclonal gammopathy of clinical significance, monoclonal gammopathy of renal significance, TEMPI syndrome, Schnitzler syndrome, thrombotic microangiopathy

Monoclonal gammopathy of clinical significance (MGCS) is an umbrella term to describe a broad spectrum of disorders with remarkable organ dysfunctions related to the underlying non-malignant B or plasma cell clone. Although the clone itself is typically very small, it is associated with diverse clinical manifestations through different mechanisms, such as monoclonal protein deposition, the biological activity of the monoclonal immunoglobulin, or angiogenic/inflammatory cytokine hyper-secretion (1, 2). Some predominantly involve a single organ, commonly peripheral nerves, kidney, or skin, while others are systemic diseases with syndromic presentations. Recognizing the clinical features with appropriate workups, in particular tissue biopsies, are the key to making a timely diagnosis, especially when the kidney or skin is affected. Treatment is often challenging and requires a multidisciplinary approach. In several conditions, immunomodulation with high-dose intravenous polyvalent immunoglobulin and/or clone-directed therapy are the best options with significant improvement of clinical symptoms and reversal of organ dysfunctions (1, 2).

Nonetheless, not every patient with an underlying monoclonal gammopathy has MGCS, given the rising prevalence of true monoclonal gammopathy of undetermined significance (MGUS) with age (3). There are no features of the serum protein electrophoresis that can distinguish MGCS from MGUS. Their heterogeneous clinical manifestations and overall rarity often make the diagnoses delayed, which could lead to devastating organ injuries. The underlying pathogenic mechanisms are complex and sometimes intertwined with several different components. Most treatment recommendations are based on anecdotal reports and retrospective experiences. Overall, more research efforts are required to better characterize these disorders from a multidisciplinary perspective, hoping to pave the way to a mechanism-driven therapeutic approach. In the current collection, seven articles are included to help improve our understanding of MGCS from different perspectives.

Monoclonal gammopathy with renal significance (MGRS) is one of the most studied disease subgroups within MGCS, and clone-directed therapy has been shown to improve or stabilize renal function deterioration in several retrospective large series focusing on kidney pathologies related to monoclonal protein deposition (4). In contrast, associations between monoclonal gammopathy and certain kidney pathologies without apparent monoclonal protein deposition are less confirmed. Filippone et al. described three patients with biopsy-proven renal-limited thrombotic microangiopathy in the context of monoclonal gammopathies. Typical primary and secondary etiologies of thrombotic microangiopathy were excluded in these patients, and clone-directed therapies were given in two of them, although a long-term follow-up is required to delineate the organ response and its correlation with the hematologic response. Da et al. reviewed the potential relationship between monoclonal gammopathy and fibrillary glomerulonephritis, and believed that it is not convincing to classify fibrillary glomerulonephritis under the subgroup of MGRS, given the overall low prevalence of monoclonal gammopathy in fibrillary glomerulonephritis, the uncertainty of prior studies to truly demonstrate the monoclonality in kidney biopsies, and the lack of robust evidence showing organ improvement in response to clone-directed therapy.

Yu et al. reported a patient with biopsy-proven immunoglobulin light chain amyloidosis-associated myopathy whose diagnosis was significantly delayed until thorough pathological examinations of the muscle biopsy. A diagnostic workflow based on a focused literature review was proposed when evaluating myopathy in a patient with a concurrent monoclonal gammopathy. Huang et al. reported their successful experience of using the Bruton’s tyrosine kinase inhibitor ibrutinib in a patient with Schnitzler syndrome associated with an underlying *MYD88* L265P-mutated IgM monoclonal gammopathy when the interleukin-1 blockade was not available, reinforcing the therapeutic role of clone-directed therapy in MGCS.

Lastly, three comprehensive review articles summarized our current understanding of TEMPI (telangiectasias, elevated erythropoietin level and erythrocytosis, monoclonal gammopathy, perinephric fluid collections, and intrapulmonary shunting) syndrome, IgM-related MGCS, and other rare MGCS. Xu et al. provided the most updated information of all published TEMPI cases to date, discussed the potential pathogenic role of macrophage migration inhibitory factor based on their recent study using whole-genome sequencing in plasma cells derived from TEMPI patients (5), and reviewed the evidence of different clone-directed therapeutic options. Girard et al. reviewed unique syndromes related to IgM monoclonal gammopathies using a clinical vignette-based approach, and the classification of IgM-related MGCS was proposed based on organ involvement. Several uncommon MGCS affecting the eye (e.g., paraproteinemic keratopathy), skin (e.g., acquired cutis laxa), and multiple systems (e.g., systemic capillary leak syndrome) were reviewed by Oganesyan et al., and the general therapeutic approaches in MGCS, namely clone-directed and immunomodulatory therapies, were emphasized based on the available evidence.

Collectively, the articles compiled on this Research Topic provided important information from various perspectives and would hopefully call for more research attention to MGCS.

## Author contributions

CW drafted the manuscript. CX and JL critically reviewed it. All authors contributed to the article and approved the submitted version.
